# Trauma-Related Guillain–Barré Syndrome: Systematic Review of an Emerging Concept

**DOI:** 10.3389/fneur.2020.588290

**Published:** 2020-11-06

**Authors:** Chuxin Huang, Yiliu Zhang, Shuwen Deng, Yijun Ren, Wei Lu

**Affiliations:** ^1^Department of Neurology, The Second Xiangya Hospital, Central South University, Changsha, China; ^2^Department of Radiology, The Second Xiangya Hospital, Central South University, Changsha, China

**Keywords:** Guillain–Barré syndrome, trauma, surgery, hemorrhage, heatstroke

## Abstract

Guillain–Barré syndrome (GBS) is mainly associated with preceding exposure to an infectious agent, although the precise pathogenic mechanisms and causes remain unknown. Increasing evidence indicates an association between trauma-related factors and GBS. Here, we performed a systematic review, summarized the current scientific literature related to the onset of GBS associated with trauma, and explored the possible pathogenesis. A literature search of various electronic databases was performed up to May 2020 to identify studies reporting diverse trauma-related triggers of GBS. Data were extracted, summarized descriptively, and evaluated with respect to possible mechanisms. In total, 100 publications, including 136 cases and 6 case series involving GBS triggered by injury, surgery, intracranial hemorrhage, and heatstroke, met our eligibility criteria. The median age of the patients was 53 [interquartile range (IQR) 45–63] years, and 72.1% of the patients were male. The median number of days between the trigger to onset of GBS symptoms was 9 (IQR 6.5–13). Overall, 121 patients (89.0%) developed post-injury/surgical GBS, whereas 13 (9.6%) and 2 (1.5%) patients had preexisting spontaneous intracranial hemorrhage and heatstroke, respectively. The main locations of injury or surgeries preceding GBS were the spine and brain. Based on available evidence, we highlight possible mechanisms of GBS induced by these triggers. Moreover, we propose the concept of “trauma-related GBS” as a new research direction, which may help uncover more pathogenic mechanisms than previously considered for typical GBS triggered by infection or vaccination.

## Introduction

Guillain–Barré syndrome (GBS) is a group of acute immune-mediated paralytic neuropathies characterized by rapidly progressive bilateral weakness of the extremities with hyporeflexia or areflexia (1). The clinical manifestations of distinctive types of GBS vary, and subtypes are mainly categorized into demyelinating and axonal forms according to the clinical course, nerve conduction velocities, and immunologic findings. The typical form of GBS is known as acute inflammatory demyelinating polyradiculoneuropathy (AIDP), whereas the main axonal forms are subdivided into acute motor axonal neuropathy (AMAN) and acute motor and sensory axonal neuropathy (AMSAN) (1–3). Miller Fisher syndrome is a less common subtype defined by the presence of anti-GQ1b antibody and can present with ophthalmoplegia, ataxia, and areflexia (4). In conjunction with supportive care, early initiation of intravenous immunoglobulins, or plasma exchange is considered crucial for treating GBS. Close respiratory monitoring is also necessary as some GBS patients may develop respiratory failure and require artificial ventilation (5).

Although the precise cause of GBS remains to be fully elucidated, a substantial proportion of diagnosed cases are associated with a history of preceding symptoms of respiratory or gastrointestinal tract infection before the onset of GBS. GBS has also been reported to develop after vaccinations. Accordingly, the majority of studies on the causes of GBS have focused on various antecedent infectious agents and vaccinations, and the understanding of these mechanisms has advanced substantially in recent years.

The development of GBS is associated with the phenomenon of molecular mimicry and with cross-reactivity. Infection can elicit cross-reactive antibodies resulting in an autoimmune attack on the peripheral nerves. Many different infectious precedents have been described in patients with GBS, with *Campylobacter jejuni* being the most frequently identified agent. The outer cell wall of *C. jejuni* contains ganglioside-mimicking lipo-oligosaccharide structures, which can induce the generation of antibodies that cross-react with specific gangliosides expressed on the peripheral nerves (3). *C. jejuni* is more likely to be associated with AMAN rather than with AIDP, and patients who develop AMAN subsequent to *C. jejuni* infection exhibit high titers of antibodies against GM1 or GD1a, which form the basis of cross-reactivity between the ganglioside complexes of peripheral motor axons and bacterial lipo-oligosaccharides (6). Upon injury, the growth-inhibiting myelin and axonal remnants from Wallerian degeneration are typically phagocytosed by resident and infiltrating macrophages of the peripheral nervous system, which are recruited by Schwann cells that induce an autophagic reaction to mediate the synthesis of chemokines and cytokines (7). Activated macrophages release inflammatory substances leading to demyelination, which can cause axonal degeneration as a secondary process (8). Various cross-reactive antibodies against gangliosides on the peripheral nerves have been reported in patients with GBS (9, 10), which are specific to different antigens that define the GBS subtype and related neurological symptoms (6).

Besides *C. jejuni*, GBS has been associated with other antecedent pathogens, including cytomegalovirus, Epstein–Barr virus, influenza virus, herpes simplex virus, adenovirus, varicella-zoster virus, *Mycoplasma pneumoniae, Haemophilus influenzae*, and most recently, severe acute respiratory syndrome coronavirus 2 (SARS-CoV-2) (11–13). Prodromal immunizations have also been proposed as a possible risk factor for GBS, although there is debate about this association owing to conflicting results among studies, and the relative risk differs among vaccination types (14, 15). GBS symptoms typically occur within a minimum of 3–5 days after immunization (16). Although the exact pathological mechanism has not been confirmed, molecular mimicry has been proposed as a trigger, along with direct damage to the myelin, or axonal membranes from the vaccine or exposure of cryptogenic epitopes (17).

Recent evidence has pointed to a role of trauma as another leading factor contributing to the pathogenesis of GBS owing to numerous reports showing a correlation between injury, surgery, hemorrhagic stroke, heatstroke, and other traumatic factors with the onset of GBS. The pathogenesis is considered to involve a variety of biochemical cascade reactions. Here, we refer to trauma in the general sense, which includes both external and internal factors leading to neurological damage that are distinct from tissue or nerve damage caused by infectious agents and immunization. The term “injury” is herein used to refer to an external injury (e.g., car accident, fall) within the broader context of trauma. Some critical patients in the intensive care unit (ICU) experience weakness of the extremities, which has traditionally been considered “ICU-acquired weakness”; however, such patients may in fact be in the early stages of trauma-induced GBS, and these symptoms will likely be ignored as such, thereby jeopardizing prompt recognition and treatment. To provide more context for this condition, we conducted a systematic review to summarize documented cases of GBS following various trauma-related triggers and discuss potential immunological and pathological mechanisms. Based on this evidence, we propose the new subtype of “trauma-related GBS” in the hope of attracting more attention and recognition to this form of GBS.

## Methods

### Data Sources

The PubMed, Web of Science, China National Knowledge Infrastructure Platform, and Wanfang electronic databases were searched up to May 1, 2020, for all studies related to GBS or variant forms associated with possible trauma-related triggers using the following search terms: “Guillain-Barré syndrome,” “AIDP,” “AMAN,” “AMSAN,” and “Miller Fisher syndrome” in combination with “trauma,” “injury,” “surgery,” “anesthesia,” “hemorrhage,” “stroke,” and “heatstroke.” We also searched the reference lists of retrieved publications to identify other potentially relevant studies.

### Selection Criteria

We followed the Preferred Reporting Items for Systematic Reviews and Meta-analyses (PRISMA) guidelines for study selection, analysis, and summary of the findings. The inclusion criteria were (1) reported trauma-related triggers before the onset of GBS and (2) articles, reviews, and case reports published in English or Chinese, with full text available. Studies reporting cases of GBS that developed following preexisting (within 6 weeks) or postoperative infection by any infectious agents (i.e., viruses, bacteria, and parasites) were excluded, such as respiratory, gastrointestinal, and urinary tract infection; superficial and deep wound infection; sepsis and septic shock; and any other systemic infectious complications. Other exclusion criteria were vaccination prior to the development of GBS within 6 weeks and lack of relevant data of reported trauma-related GBS cases to permit analysis.

### Data Extraction and Synthesis of Results

Each study was subject to a detailed review, during which the following information was extracted: possible triggers, sex, age, duration from triggers to onset of symptoms, elevated protein, or albumin-cytological dissociation in the cerebrospinal fluid (CSF), treatment, respiratory support, and mortality. As some reports of GBS patients who had experienced traumatic brain injury failed to document the severity and whether or not there was bleeding, we limited our analysis to focus on cases of spontaneous hemorrhagic stroke within the intracranial hemorrhage group. Data on the characteristics, clinical presentation, laboratory tests, and outcomes were summarized as median and interquartile range (IQR) or proportion, as appropriate. Some of the included studies provided information that was unclear or incomplete, including days from triggers to onset, CSF parameters, conditions of respiratory support, and prognosis; such data were thereby excluded, and corresponding calculation of numbers and frequencies was only performed for studies with complete data. Owing to the heterogeneity among the included cases, along with the exclusion of trauma-related GBS cases with preceding infection or incomplete data, we focused on describing the results and synthesizing them in a qualitative manner. For six case series (with aggregated data) including post-injury/postsurgery GBS patients, the detailed information could not be extracted for analysis; hence, we summarized the total number of patients, subject characteristics, and main conclusions of each study.

## Results

**Figure 1:**
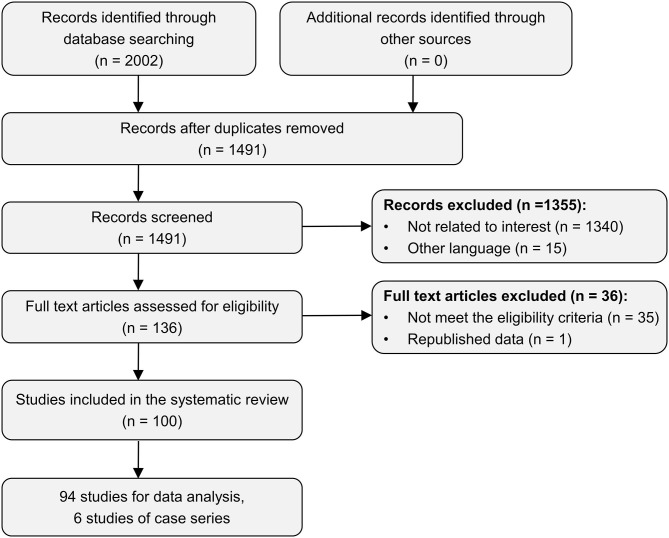
PRISMA flow diagram of the selection procedure of included studies in the systematic review.

A total of 100 publications (7, 14, 15, 18–114) met the eligibility criteria for the review, 94 of which (7, 14, 18–109) included suitable data for analysis. Ninety-four studies involved a total of 136 patients diagnosed with GBS associated with trauma-related triggers, including injury, surgery, intracranial hemorrhage, and heatstroke, which were subjected to analysis (Table 1). Six case series (15, 110–114) including trauma-related GBS patients with aggregated data were reviewed and generalized (Table 2). Figure 1 shows the PRISMA flow diagram of the selection procedure of included studies.

**Table 1:** Demographic and clinical characteristics of patients with trauma-related Guillain–Barré syndrome or variant forms.

**Trauma-related triggers**	**No. of patients, *n***	**Male sex, *n* (%)**	**Age, y, median (IQR)**	**Duration from triggers to onset of symptoms, d, median (IQR)^†^**	**Elevated protein/albumin-cytological dissociation in CSF, *n* (%)^†^**	**Treatment**, ***n*** **(%)**^**†**^						**Respiratory support, *n* (%)^†^**	**Mortality, *n* (%)^†^**	**References**
						**IVIg**	**Plasmapheresis**	**Steroids**	**IVIg + plasmapheresis**	**IVIg + steroids**	**Conservative treatment**			
**Injury and surgery**	121	88 (72.7)	54 (45–63)	9 (6.75–13)	77 (92.8)	51 (48.1)	17 (16.0)	8 (7.5)	7 (6.6)	11 (10.4)	9 (8.5)	54 (50.9)	7 (6.4)	–
Brain injury	29	21 (72.4)	47 (39–58)	10 (7–12)	22 (100.0)	15 (55.6)	5 (18.5)	–	–	3 (11.1)	4 (14.8)	14 (56.0)	2 (7.4)	(7, 18–40)
Brain surgery^#^	10	5 (50.0)	56 (48.5–73.25)	9.5 (6.75–11.75)	7 (100.0)	3 (37.5)	2 (25.0)	1 (12.5)	1 (12.5)	1 (12.5)	–	5 (50.0)	1 (12.5)	(23, 24, 29, 39–44)
Oral maxillofacial	3	1 (33.3)	55 (37–63.5)	12 (9.5–66)	0 (0)	–	–	–	–	–	1 (100.0)	1 (33.3)	0 (0)	(45, 46)
Spinal^‡^	33	23 (69.7)	53.0 (47–62)	8 (3–12)	21 (100.0)	16 (50.0)	3 (9.4)	3 (9.4)	4 (12.5)	3 (9.4)	–	14 (51.9)	1 (3.1)	(14, 29, 38, 47–68)
Cardiac	10	9 (90.0)	62 (54.5–65.75)	10.5 (3.5–13.5)	6 (75.0)	5 (50.0)	4 (40.0)	–	1 (10.0)	–	–	7 (70.0)	1 (10.0)	(40, 69–75)
pulmonary surgery and chest injury	4	3 (75.0)	56 (48.5–59.5)	10.5 (7.75–11.75)	3 (75.0)	1 (25.0)	1 (25.0)	–	–	1 (25.0)	1 (25.0)	3 (75.0)	1 (25.0)	(23, 38, 76, 77)
Abdominal	17	14 (82.4)	62 (50–67)	7 (4–10)	10 (83.3)	5 (41.7)	1 (8.3)	4 (33.3)	1 (8.3)	–	1 (8.3)	6 (40.0)	1 (7.1)	(14, 24, 78–89)
Orthopedic (pelvis and extremities)	9	6 (66.7)	52 (45–64)	12 (7–16)	4 (100.0)	5 (62.5)	1 (12.5)	–	–	1 (12.5)	1 (12.5)	4 (50.0)	0 (0)	(14, 23, 53, 90–95)
Brachial plexus	1	1 (100.0)	51 (–)	15 (–)	1 (100.0)	–	–	–	–	–	1 (100.0)	0 (0)	0 (0)	(96)
Polytrauma	3	3 (100.0)	55 (42.5–57)	17 (15–17)	1 (100.0)	1 (100.0)	–	–	–	–	–	0 (0)	0 (0)	(14, 97)
Electrical injury	2	2 (100.0)	35 (27–43)	10 (8.5–11.5)	2 (100.0)	–	–	–	–	2 (100.0)	–	0 (0)	0 (0)	(98, 99)
**Spontaneous intracranial hemorrhage**^**§**^	13	8 (61.5)	51 (42–58)	10 (8–14)	11 (100.0)	7 (53.8)	1 (7.7)	–	1 (7.7)	2 (15.4)	–	6 (54.5)	0 (0)	(28, 29, 39, 100–107)
**Heatstroke**	2	2 (100.0)	34.5 (31.25–37.75)	7.5 (6.25–8.75)	2 (100.0)	–	–	–	1 (50.0)	1 (50.0)	–	0 (0)	0 (0)	(108, 109)
Total	136	98 (72.1)	53 (45–63)	9 (6.5–13)	90 (93.8)	58 (47.9)	18 (14.9)	8 (6.6)	9 (7.4)	14 (11.6)	9 (7.4)	60 (50.4)	7 (5.6)	–

‡ *IQR, interquartile range; IVIg, intravenous immunoglobulin; NA, not available*.

† *Some of the included studies provided information that was unclear or incomplete, and thus corresponding calculation of numbers and frequencies were only performed for studies with complete data*.

# *In this group, the surgical operations were not induced by external injury. In this group, 3 patients received IVIg + steroids + plasmapheresis treatment*.

§ *In this group, one patient received plasmapheresis together with steroids, and one patient received IVIg + steroids + plasmapheresis*.

**Table 2:** Summary of case series investigating the association of Guillain–Barré syndrome (GBS) with trauma-related triggers.

**Study**	**Total population studied/patients with trauma-related GBS**	**Comments**
Sipila et al. (15)	GBS patients with preceding triggers including infection, vaccination, and surgery (*n* = 69) Patients with post-surgical GBS (*n* = 4)	The most common triggers among overall incidence of GBS were respiratory tract infections and gastroenteritis, while surgery was a relatively rare risk factor.
Guan et al. (110)	Patients with surgery-related GBS (*n* = 10)	GBS should be considered in patients after surgery, especially in patients with quadriplegia after limb or lumbar surgery, within 2 weeks.
Rudant et al. (111)	GBS patients (*n* = 8,364) Patients with post-surgical GBS in the referent (*n* = 175) and case windows (*n* = 257)	GBS was moderately associated with any type of recent surgery and was more strongly associated with bone and digestive organ surgery.
Hocker et al. (112)	GBS patients (*n* = 208) Patients with post-surgical GBS (*n* = 19)	Surgery antedated GBS in 9.1% of patients, and post-surgical GBS was more common in patients with an active malignancy.
Zhu et al. (113)	GSB patients with preceding triggers including infection and trauma (*n* = 51) Patients with trauma-related GBS (*n* = 17)	Compared with infection-related GBS, trauma-related GBS is associated with patient characteristics of older age, a high proportion of axonal damage, and more severe symptoms and prognosis. Furthermore, there was no additional correlation identified between anti-ganglioside antibody and trauma-related GBS.
Bao et al. (114)	Patients with post-surgical GBS (*n* = 17)	Post-surgical GBS patients often exhibit axonal subtypes of GBS with severe motor dysfunction, high risk of respiratory failure, and poor prognosis.

In the analysis, the median age of the study population was 53 (IQR 45–63) years, with a 2.58 male-to-female ratio (72.1% men and 27.9% women). The median duration from the trauma trigger to onset of the first GBS symptom was 9 (IQR 6.5–13) days. Overall, 121 of the 136 patients (89.0%) had undergone injury and/or a surgical procedure before the onset of GBS, whereas only 13 (9.6%) and 2 (1.5%) patients had preexisting spontaneous intracranial hemorrhage and heatstroke, respectively. The spine (33 patients, 27.3%) was the most common location of injury or surgery described, followed by traumatic brain injury (29 patients, 24.0%).

CSF was collected in most cases for analysis, with elevated protein levels and/or albumin-cytological dissociation observed in 93.8% of cases. Anti-ganglioside antibodies in the serum or CSF as well as the subtype of GBS were only described in 37 cases; thus, these incomplete data were not further analyzed.

The most common treatment administered was immunoglobulins in 58 patients (47.9%) and plasmapheresis in 18 patients (14.9%), while 9 patients (7.4%) received both. Overall, 60 patients (50.4%) received artificial ventilation, and only 7 patients (5.6%) who had experienced injury or surgery died during the follow-up period.

## Discussion

### Trauma-Related Triggers of GBS and Underlying Mechanisms

#### Injury

GBS following multiple types of injuries has been increasingly reported in recent years. Although the underlying pathophysiological mechanisms of the association between injury and GBS has not yet been completely elucidated, we consider that injury-related GBS may involve various biochemical cascades and immunologic responses resulting from psychological stress, physical damage, and pain. In particular, there is accumulating evidence that an increase of heat shock protein 70-kDa (HSP70) may play a role in the pathogenesis. Here, we collectively refer to an external injury (e.g., car accident, fall) as “injury” to distinguish from other sources of trauma.

First, the impact of psychological stress resulting from injury on the development of GBS has been recognized, although its specific role in GBS pathogenesis remains inadequately understood. Psychological stress has been associated with alteration of the immune system. Anghelescu et al. (115) suggested that this response is most likely due to complex interactions between the neuro-endocrine-immune pathways, in which the primary mediators include hormones of the hypothalamic–pituitary–adrenal axis, catecholamines, and cytokines. To maintain homeostasis in a condition of stress, corticotropin-releasing hormone can be stimulated, which subsequently induces the release of adrenocorticotropic hormone as well as the downstream factors adrenaline, noradrenaline, and cortisol (115). Catecholamines mediate immune responses by promoting the expression of cytokines such as interleukin (IL)-6, IL-8, IL-1β, and tumor necrosis factor-alpha (TNF-α), which were also reported to be involved in the pathogenesis of GBS (116, 117). Esposito et al. (118) reported that acute stress can play a pro-inflammatory role by activating mast cells, leading to the release of several vasoactive mediators, thereby increasing permeability of the blood–brain barrier (BBB) in rats. Auto-reactive leukocytes and some potential mediators in the central nervous system are considered to be transported through the disrupted BBB to the peripheral nervous system, ultimately causing damage to the myelin and axons. There is some evidence that Toll-like receptor 4 (TLR4) plays a crucial role in the stress-induced immune and inflammatory response. Zhang et al. (119) reported that stress-mediated T-helper (Th)1 cytokine suppression and Th2-induced cytokine increase were significantly reduced in TLR4-deficient mice compared with those in wild-type mice with normal TLR4 expression. Similarly, another study found that TLR4-deficient mice exposed to subacute stress had better behavioral performance compared to wild-type mice (120). In addition, the mRNA level of *TLR4* was reported to be significantly increased in GBS patients compared with that in healthy controls (121). Accordingly, TLR4 may act as a mediator between psychological stress and the pathogenesis of GBS. Although it has not been reported to date, GBS following psychological stress deserves further attention to help predict GBS onset.

Second, physical damage induces alterations in immunological status and systemic inflammatory responses mediated by a variety of molecules. Roberts et al. (122) revealed an elevation of matrix metalloproteinases (MMPs) in the microdialysate of patients with severe traumatic brain injury. MMPs can facilitate the adhesion to and transmigration across the BBB of immune cells, including macrophages, which can secrete a diverse range of neurotoxic mediators such as TNF-α and nitric oxide to ultimately cause damage to the axons and myelin (123). TNF-α can also enhance chemokine ligand 2 and intercellular adhesion molecule 1 in peripheral nerve microvascular endoneurial endothelial cells (124). In particular, in response to traumatic brain injury, both the original insult and the secondary activation of inflammatory cascades involving the expression of cytokines, chemokines, and MMPs can result in impairment of the BBB, allowing for the migration and accumulation of immune cells, especially leukocytes, from the central nervous system (125, 126). Moreover, Hang et al. (127) demonstrated that nuclear factor (NF)-κB can be activated after traumatic brain injury and subsequently induce the expression of inflammatory cytokines such as IL-6 and TNF-α. TLR-4 also appears to have a functional role in this process. After experimental brain injury, TLR4-deficient mice had fewer brain lesions and reduced production of inflammatory molecules than wild-type mice (128). The aforementioned inflammatory molecules have been proposed to be associated with the development of GBS (117). Thus, we consider that they may function as mediators between physical damage and GBS pathogenesis.

Third, tissue injury and inflammation are usually accompanied by pain, and the perception of pain is mediated by nociceptors. Immune-related cytokines such as IL-1β, IL-6, TNF-α, and IL-17A bind to their respective receptors on nociceptors, leading to the generation of pain impulses (129). These cytokines have also been established to play important roles in GBS as described above (117).

Fourth, injury can cause the production of HSPs, among which HSP70 has been identified to be elevated in the CSF of GBS patients and is likely involved in the pathogenesis of GBS development. HSPs are a class of polypeptides that participate in immune regulation and stress resistance and may also contribute to mechanisms of autoimmune inflammatory diseases. Upon cellular injury, endogenous HSP70 is released in the extracellular compartment to enhance antigen presentation and stimulate pro-inflammatory responses. Extracellular HSP70 may thereby serve as a molecular link between injury, systematic inflammatory cascades, and subsequent GBS development. HSP70 acts as a molecular chaperone to bind antigens and mediate their internalization to antigen-presenting cells (APCs). After being taken up by APCs, the HSP–antigen complexes activate antigen cross-presentations through major histocompatibility complex (MHC) class I or MHC class II pathways to consequently activate the T-cell response. In this process, intracellular and exogenous antigens appear to be processed by MHC class I molecules in the proteasome or MHC class II molecules in lysosomes, and the peptides are subsequently presented to CD8+ T cells and CD4+ T cells, respectively, which exert diverse immunomodulatory effects (130). Moreover, extracellular HSP70 can elicit the activation of HSP70 receptor and its co-receptor CD14. The binding of HSP70 to the plasma membrane of human monocytes elicits a rapid intracellular calcium flux, leading to NF-κB activation and the subsequent transcription and production of the pro-inflammatory cytokines TNF-α, IL-1β, and IL-6. This process involves two different signal transduction cascades: one dependent on CD14 and intracellular calcium, leading to an increase of IL-1β, IL-6, and TNF-α, and the other independent of CD14 but dependent on intracellular calcium, resulting in increased TNF-α instead of IL-1β or IL-6 (131). Extracellular HSP70 can also promote further activation of the inflammasomes (132).

In a proteomics analysis of CSF from GBS patients, HSP70 expression was found to be significantly upregulated (133). Another study measured 10 HSP antibody concentrations in the sera and CSF from patients with GBS, demonstrating higher immunoglobulin (Ig)G antibody titers against the HSP27, HSP60, HSP70, and HSP90 families in the CSF than those of patients with motor neuron disease (134). Helgeland et al. (135) demonstrated higher levels of total Ig (IgG, IgA, and IgM) in the sera of GBS patients than those of healthy controls. The pathological role of HSP70 antibodies in GBS patients and the association between antibody titers and clinical features remains unclear. However, these findings may reflect ongoing autoimmune disease activity in the peripheral nervous system, offering research directions for the further exploration of candidate biomarkers of GBS. Moreover, HSPs can function as markers of reversible cellular injury. Hashiguchi et al. (136) reported significantly elevated expression levels of HSP27, HSP60, HSP70, and HSP90 in polymorphonuclear leukocytes of 50 patients with severe injuries compared with those of 17 healthy volunteers in all three periods examined (days 0–1, days 2–5, and days 6–14) after trauma events (136). The induction of HSP27 and HSP72 has also been reported to differ with respect to both location and time points following mild or severe injuries of the cerebellum (137). These studies on HSPs may partly explain the relationship between injuries and GBS.

αB-Crystallin (αBC) is a small HSP with a cytoprotective function, and its expression is upregulated under a state of cellular stress caused by various injuries (138, 139). Wanschitz et al. (139) compared the anti-αBC-IgG antibody level in patients with GBS, infectious inflammatory neurological diseases, multiple sclerosis, and non-inflammatory neurological disorders and demonstrated increased αBC-IgG indices in a high proportion of GBS patients (139). Elevated αBC-IgG might therefore represent a mechanistic link between injuries and GBS pathogenesis. Cathepsin B is a major lysosomal cysteine protease for intracellular protein catabolism in neurons and plays crucial roles in cell death after neuronal injury (140). Cystatin C, which is known as a cysteine protease inhibitor, is tightly bound to cathepsin B in the CSF (141). A decrease in the cystatin C expression level and high cathepsin B enzymatic activity have been observed in patients with GBS and chronic inflammatory demyelinating polyneuropathy compared with those of control subjects. Thus, cystatin C and cathepsin B may also be involved in the pathogenesis of injury-related GBS (141).

#### Surgery

Surgery, particularly brain and spinal surgery, is a major contributor to trauma-related GBS, although the details of the pathophysiology remain to be clearly defined. We consider the following mechanisms to be most likely. First, surgery-associated GBS can also lead to psychological stress, pain, physical damage, and disruption of the BBB, resulting in the pathogenic pathways described in the section above. Although surgeries of locations other than the brain and spine do not result in direct BBB damage, some degree of BBB disruption may nevertheless occur. Merino et al. (142) reported magnetic resonance imaging-detected BBB impairment in nearly half of 19 patients after cardiac surgery in a case series. Moreover, Wang et al. (143) found that BBB permeability and the level of tight-junction proteins increased following splenectomy in rats. Activation of the systemic inflammatory response can be one of the mechanisms leading to breakdown of the BBB after different surgeries (144), and immune cells moving from the central nervous system to the peripheral nervous system via the disrupted BBB release various cytokines, leading to destruction of the myelin sheath, and axons. Second, the expression of HSPs, especially HSP70, and numerous cytokines can be altered during surgical procedures; thus, postsurgical GBS may share the same pathogenesis with post-injury GBS. Third, deterioration of preexisting subclinical inflammation and mechanical forces may also contribute to surgery-related GBS (82, 145).

The use of anesthetics before surgery may also be responsible for the subsequent onset of GBS, and several hypotheses have been proposed to explain this phenomenon, including maldistribution of local anesthetics, surgical positioning, and pharmacologic agent chemical irritation or neurotoxicity, resulting in breakdown of the blood–nerve root barrier by a non-infectious inflammatory process (79, 146). Therefore, we do not consider anesthesia to be a trigger of trauma-related GBS as the mechanism appears to be different.

#### Intracranial Hemorrhage

GBS has also been reported to occur following spontaneous intracranial hemorrhage, which includes subarachnoid, subdural, and cerebral hemorrhages, and hemorrhagic transformation of ischemic stroke. An association between intracranial hemorrhage and GBS has been proposed, either in a spontaneous or in posttraumatic context. However, the exact pathogenesis of GBS secondary to intracranial hemorrhage remains to be further explained.

GBS following spontaneous intracranial hemorrhage or traumatic brain injury have an overlap of etiological mechanisms, as discussed in detail above. On the one hand, immune cells originating from the central nervous system are transported through the impaired BBB to the peripheral nervous system where they release certain mediators to cause demyelination or axonal damage. On the other hand, intracranial hemorrhage can further induce the production of HSPs, especially HSP70, as candidate biomarkers of GBS.

Haptoglobin has also been reported as a potential biomarker of GBS, which plays an important regulatory role in cerebral hemorrhage; therefore, we postulated that haptoglobin may serve as a mediator in the pathogenic mechanism of GBS following intracranial hemorrhage. When cerebral blood vessels and brain parenchyma are impaired following cerebral hemorrhage, erythrocyte lysis occurs, leading to extracellular infiltration of hemoglobin, heme, and iron in the central nervous system to induce secondary brain injury (125). To offset the process of neuronal damage arising from intracranial hemorrhage, various pathways, including those involving phagocytic cells, are activated to remove the hemoglobin and its breakdown products. The predominant pathway for hemoglobin clearance is the CD163–haptoglobin–hemoglobin system (147). Haptoglobin, an acute-phase response glycoprotein, binds to and neutralizes circulating hemoglobin in the bloodstream and is expressed in the central nervous system during intracranial hemorrhage to minimize toxic damage (148). Haptoglobin is mainly synthesized by hepatocytes and the reticuloendothelial system and diffuses into the CSF from the circulating blood. After intracranial hemorrhage, hemoglobin can be captured by haptoglobin, and the bound complex is recognized and taken up by CD163, as a membrane scavenger receptor, to initiate endocytosis by macrophages and microglia (147).

Galea et al. (149) analyzed CSF from patients following subarachnoid hemorrhage, demonstrating free extracellular hemoglobin despite detectable haptoglobin within the intrathecal compartment. This is related to the lower haptoglobin hemoglobin-binding capacity of the central nervous system than that of the circulation; thus, the intrathecal hemoglobin-scavenging system is saturated in subarachnoid hemorrhage, thereby driving a large amount of hemoglobin efflux out of the central nervous system across the BBB to rely on peripheral hemoglobin scavenging.

The serum haptoglobin levels of both GBS pretreatment patients and patients with aseptic meningitis were reported to be elevated compared with those of healthy controls. However, the larger (nearly 4-fold) increase in haptoglobin levels in pretreatment GBS patients indicated that haptoglobin might be a crucial mediator in GBS pathology, instead of a reflection of a generalized acute-phase response (150). Among the candidate biomarkers identified by Li et al. (133) in a proteomics analysis, haptoglobin was found to be overexpressed in the CSF of GBS patients, thus supporting our speculation that intracranial hemorrhage may serve as a trigger in the development of GBS. To the best of our knowledge, there has been no report of GBS following ischemic stroke, and the involvement of haptoglobin in distinguishing the pathogenesis of secondary complications related to hemorrhagic and ischemic stroke warrants further study.

#### Heatstroke

Heatstroke is a severe illness with a common feature of hyperthermia and failure to dissipate excessive body heat. Heatstroke reduces intestinal blood flow to result in ischemia, which in turn increases gastrointestinal permeability. The consequent oxidative stress disrupts cell membranes and tight junctions, triggering alterations in immune status, and stimulating macrophages to release cytokines such as TNF-α, IL-1, IL-6, and interferon-gamma, which adversely affect permeability of the blood–nerve barrier (108, 151). As a result, myelinotoxicity of the cytokines may cause damage to the myelin sheath and contribute to the development of GBS. Moreover, heatstroke can induce the production of HSPs which appears to be related with the mechanism of subsequent GBS development as described above.

#### Strenuous Exercise, Excessive Fatigue, and Cardiac Arrest

We recently experienced three cases of GBS following strenuous exercise or excessive fatigue (unpublished data), which are associated with strenuous physical activities in which excessive production of metabolic heat overwhelms physiological heat-loss mechanisms (151). In addition, we experienced a case of cardiac arrest inducing GBS. To our knowledge, these triggers have not been reported in association with GBS previously.

#### Other Factors

Some other trauma-related factors have been reported to be of temporal relevance to GBS, but no specific mechanism has been identified thus far. Accordingly, further research in this field should focus on whether the subsequent GBS is directly related to trauma, caused by other mechanisms, or their combination. Overall, the relevance of other types of trauma to GBS remains controversial; these include bites or stings of animals (152, 153); suffering from malignant diseases (154); transplantation of stem cells, bone marrow, or solid organs (liver and kidney) (155); administration of immunosuppressive drugs and anticancer agents (156); comorbid autoimmune diseases, including systemic lupus erythematosus (157) and multiple sclerosis (158); myocardial infarction (159); and bariatric surgical procedures for morbid obesity performed through gastrectomy and gastric bypass (160), alcohol consumption (161), and heroin addiction (162).

GBS can also be secondary to pregnancy, with variation in the onset time. An increased incidence of GBS in the third trimester and first 2 weeks after delivery was reported in a case series of 47 pregnant patients (163). GBS diagnosed during pregnancy may be aggravated in the postpartum period because of an increase in a delayed type of hypersensitivity (164). Rolfs et al. (165) considered that cell-mediated immunity may be the primary mechanism in the pathogenesis of GBS with pregnancy, since humoral factors are shared by mothers and infants through passive transfer whereas cellular components are not. Fetal cells moving into the maternal circulation and endogenous antigen transfer could play a role in this mechanism (163).

### Limitations

Some limitations of the study should be addressed. First, a certain degree of bias may have been introduced in the analysis owing to the heterogeneity in the diagnosis and treatment procedures and description details of different cases, along with the exclusion of trauma-related GBS cases with preceding infection or incomplete data. Thus, we focused on a qualitative analysis to discuss the possible mechanisms of different trauma-related triggers. Second, studies published in languages other than English and Chinese were not included, and therefore we may have missed relevant cases that were reported in other languages. Third, some other confounding factors interacting with trauma-related triggers may ultimately cause the development of GBS, which were not described herein. Therefore, more large-scale clinical studies and further basic research are needed.

## Conclusion and Future Perspectives

**Figure 2:**
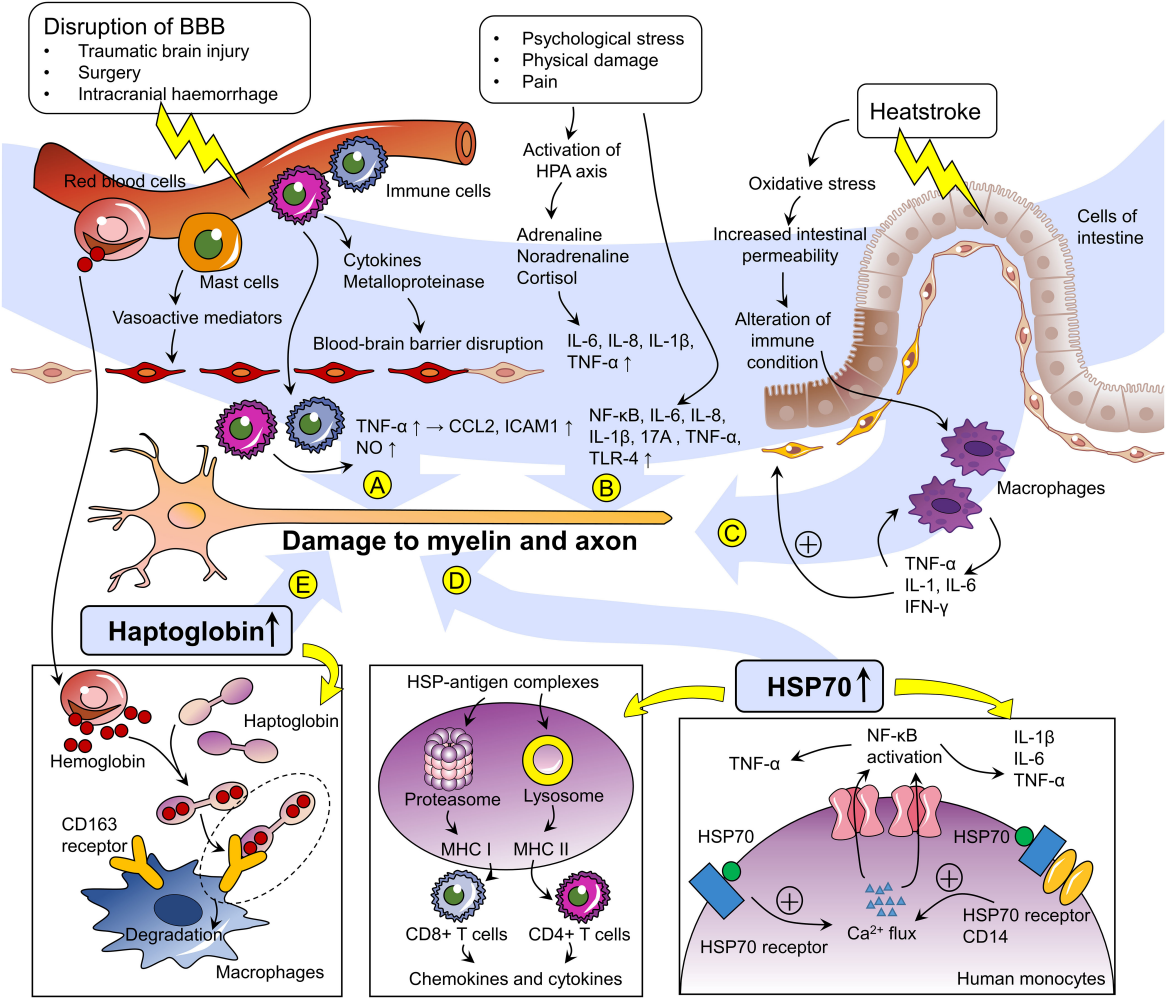
Proposed mechanisms of trauma-related Guillain–Barré syndrome. **(A)** Impairment of cerebral blood vessels induces inflammatory cascades involving the expression of cytokines and metalloproteinases, resulting in disruption of the blood–brain barrier (BBB). Mast cells in the brain also release vasoactive mediators regulating the permeability of the BBB. Immune cells thereby transmigrate across the BBB and release pro-inflammatory molecules such as NO and TNF-α. The latter enhances the expression of CCL2 and ICAM-1 in peripheral nerve microvascular endoneurial endothelial cells. The aforementioned alteration of inflammatory molecule expression may cause damage to myelin and axons in the peripheral nerves. **(B)** Psychological stress, physical damage, and pain related to the processes of injury and trauma induce activation of the hypothalamus–pituitary axis (HPA) and increase the expression levels of a wide range of factors, including NF-κB, IL-6, IL-8, IL-1β, IL-17A, TNF-α, and TLR-4, which have been proposed to be involved in the pathogenesis of GBS. **(C)** Heatstroke induces oxidative stress and increases intestinal permeability, thereby triggering alterations in immune status and stimulating macrophages to release TNF-α, IL-1, 6, and IFN-γ, which may possess myelin toxicity. **(D)** Extracellular HSP70 induced by stress serves as a molecular chaperone binding antigens and mediating their internalization into antigen-presenting cells (APCs). After being taken up by APCs, the HSP–antigen complexes activate antigen cross-presentations through MHC class I molecules in the proteasome or MHC class II molecules in lysosomes, and the peptides are subsequently presented to CD8+ T cells and CD4+ T cells, respectively. HSP70 can also elicit activation of the HSP70 receptor and the co-receptor CD14. Binding of HSP70 to the plasma membrane of human monocytes elicits a rapid intracellular calcium flux, leading to NF-κB activation and subsequent transcription and production of the pro-inflammatory cytokines TNF-α, IL-1β, and IL-6. **(E)** After cerebral hemorrhage, erythrocyte lysis, and extracellular infiltration of hemoglobin occur. To offset the process of neuronal damage, hemoglobin is captured by haptoglobin, and this complex is recognized by the membrane scavenger receptor CD163 to initiate endocytosis by macrophages. Haptoglobin has also been reported as a potential biomarker of GBS. NO, nitrogen monoxide; TNF-α, tumor necrosis factor-alpha; CCL2, chemokine ligand 2; ICAM-1, intercellular adhesion molecule-1; NF-κB, nuclear factor kappa-B; IL, interleukin; TLR4, Toll-like receptor 4; IFN-γ, interferon-γ; HSP70, heat shock protein 70-kDa; MHC, major histocompatibility complex.

Overall, this review demonstrates that a variety of trauma-related factors, including injury, surgery, intracranial hemorrhage, heatstroke, high-intensity exercise, or excessive fatigue, and cardiac arrest, may be associated with the onset of GBS. Although the specific pathogenesis remains to be further clarified, we have summarized the proposed mechanism in Figure 2. According to our experience, this type of GBS does not appear to be as rare as previously considered. Nevertheless, given the current focus on infectious agents as the main cause of GBS, trauma may be neglected as a potential cause of GBS, leading to misdiagnosis or missed diagnosis in clinical practice. The proposal of the new concept of “trauma-related GBS” highlighted herein can help bring focus to this less common type of GBS to improve recognition, understanding, and appropriate treatment.

## Data Availability Statement

The original contributions presented in the study are included in the article/supplementary materials, further inquiries can be directed to the corresponding author/s.

## Author Contributions

WL conceived of the study. SD and YR selected reports and extracted the data. CH and YZ analyzed and interpreted the data. CH and WL drafted the manuscript. All authors approved the final version. All authors contributed to the article and approved the submitted version.

## Conflict of Interest

The authors declare that the research was conducted in the absence of any commercial or financial relationships that could be construed as a potential conflict of interest.
